# Novel Mechanisms of Tumor Promotion by the Insulin Receptor Isoform A in Triple-Negative Breast Cancer Cells

**DOI:** 10.3390/cells10113145

**Published:** 2021-11-12

**Authors:** Veronica Vella, Marika Giuliano, Alessandro La Ferlita, Michele Pellegrino, Germano Gaudenzi, Salvatore Alaimo, Michele Massimino, Alfredo Pulvirenti, Alessandra Dicitore, Paolo Vigneri, Giovanni Vitale, Roberta Malaguarnera, Andrea Morrione, Andrew H. Sims, Alfredo Ferro, Marcello Maggiolini, Rosamaria Lappano, Ernestina Marianna De Francesco, Antonino Belfiore

**Affiliations:** 1 Endocrinology Unit, Department of Clinical and Experimental Medicine, University of Catania, Garibaldi-Nesima Hospital, 95122 Catania, Italy; veronica.vella@unict.it (V.V.); giulianomarika92@gmail.com (M.G.); ernestinamarianna@yahoo.it (E.M.D.F.); 2 Bioinformatics Unit, Department of Clinical and Experimental Medicine, University of Catania, 95131 Catania, Italy; alessandrolf90@hotmail.it (A.L.F.); salvatore.alaimo@unict.it (S.A.); alfredo.pulvirenti@unict.it (A.P.); alfredo.ferro@unict.it (A.F.); 3 Department of Cancer Biology and Genetics, The Ohio State University, Columbus, OH 43210, USA; 4 Department of Pharmacy, Health and Nutritional Sciences, University of Calabria, 87036 Rende, Italy; michele.pellegrino@unical.it (M.P.); marcellomaggiolini@yahoo.it (M.M.); rosamaria.lappano@unical.it (R.L.); 5 Laboratory of Geriatric and Oncologic Neuroendocrinology Research, Istituto Auxologico Italiano, IRCCS, 20095 Cusano Milanino, Italy; germano.gaudenzi@gmail.com (G.G.); alessandra.dicitore@libero.it (A.D.); giovanni.vitale@unimi.it (G.V.); 6 Oncology Unit, Department of Clinical and Experimental Medicine, University of Catania, 95124 Catania, Italy; michedot@yahoo.it (M.M.); vigneri.p@unict.it (P.V.); 7 Department of Medical Biotechnology and Translational Medicine, University of Milan, 20122 Milan, Italy; 8 Faculty of Medicine and Surgery, Kore University of Enna, 94100 Enna, Italy; roberta.malaguarnera@unikore.it; 9 Sbarro Institute for Cancer Research and Molecular Medicine and Center for Biotechnology, Department of Biology, College of Science and Technology, Temple University, Philadelphia, PA 19122, USA; andrea.morrione@temple.edu; 10 MRC Institute of Genetics and Molecular Medicine, University of Edinburgh, Scotland EH4 2XR, UK; andrew.sims@ed.ac.uk

**Keywords:** insulin receptor isoform A, insulin receptor isoforms, IGF axis, breast cancer, triple negative breast cancer, insulin receptor isoform transcriptome, tumor promotion, hyperinsulinemia

## Abstract

The insulin receptor isoform A (IR-A) plays an increasingly recognized role in fetal growth and tumor biology in response to circulating insulin and/or locally produced IGF2. This role seems not to be shared by the IR isoform B (IR-B). We aimed to dissect the specific impact of IR isoforms in modulating insulin signaling in triple negative breast cancer (TNBC) cells. We generated murine 4T1 TNBC cells deleted from the endogenous insulin receptor (*INSR*) gene and expressing comparable levels of either human IR-A or IR-B. We then measured IR isoform-specific in vitro and in vivo biological effects and transcriptome in response to insulin. Overall, the IR-A was more potent than the IR-B in mediating cell migration, invasion, and in vivo tumor growth. Transcriptome analysis showed that approximately 89% of insulin-stimulated transcripts depended solely on the expression of the specific isoform. Notably, in cells overexpressing IR-A, insulin strongly induced genes involved in tumor progression and immune evasion including chemokines and genes related to innate immunity. Conversely, in IR-B overexpressing cells, insulin predominantly induced the expression of genes primarily involved in the regulation of metabolic pathways and, to a lesser extent, tumor growth and angiogenesis.

## 1. Introduction

Breast cancer (BC) accounts for approximately 25% of all cancers and 15% of all cancer deaths in women. Progression to metastatic spread and resistance to chemotherapeutic drugs are major factors involved in BC-related mortality [1]. Notably, hyperinsulinemia is a major contributor to BC progression and metastatic dissemination [2]. Hyperinsulinemia is common in patients affected by obesity, a condition that has more than doubled in the past 30 years, reaching 40% in the United States and 30% in Europe [3]. In dysmetabolic and hyperinsulinemic obese patients, BC is often resistant to conventional and targeted therapies, it metastasizes faster and has worse prognosis. Notably, approximately 80% of BCs overexpress the insulin receptor (IR) [4]. Moreover, constitutive IR autophosphorylation is associated with high BC mortality [5]. A key insight towards a better understanding of the role of the IR in BC came from the discovery that BC often overexpresses the IR isoform A (IR-A), also known as the ‘oncofetal’ IR isoform [6]. The IR-A is generated by alternative splicing involving the skipping of exon 11 of the insulin receptor (*INSR*) gene and differs from the full-length IR-B isoform by lacking 12 amino acids. While the IR-B is considered the major physiological mediator of insulin-dependent metabolic actions, the IR-A plays an increasingly recognized role in fetal growth and tumor biology [4]. In fact, it regulates several aspects of tumor progression, including metabolic reprogramming [7], cell invasion, metastasis, epithelial-to-mesenchymal transition (EMT), stem-like cell phenotype, and resistance to cancer therapies [4]. IR-A is not exclusively expressed in fetal and tumor cells. It is instead also co-expressed with the IR-B in most healthy cells, except liver. In adult life IR isoforms’ relative abundance is strictly regulated in a tissue-specific manner with IR-A being generally predominant in non-classical insulin targets as brain and immune cells [4]. However, the role of IR-A has been mostly studied in development and in cancer, and its physiological role awaits further elucidation [4]. In addition, the mechanisms regulating IR-A-dependent pro-tumorigenic actions are still poorly characterized. 

The finetuned differences in intracellular signaling mediated by the two IR isoforms [8] can be partially explained by their putative association with different membrane subdomains, different kinetics of receptor trafficking [9], and differential interactions with downstream molecular partners [10,11]. Moreover, the IR-A is the bona fide high-affinity receptor for IGF2, which induces biased and more potent mitogenic signals than insulin [12]. IR-A overexpression has been established as a mechanism of cancer resistance to target therapies with anti-IGF1R antibodies [13,14]. Collectively, these results strongly suggest that specifically targeting the IR-A in BC and other malignancies might be an attractive approach to therapy. However, this approach remains a challenging task considering that the two IR isoforms differ by only 12 amino acids and IR-B inhibition should be avoided to prevent insulin resistance and consequent hyperinsulinemia and diabetes mellitus. Tools specifically targeting the IR-A are in fact currently unavailable [7]. 

Triple-negative breast cancers (TNBC) are an heterogenous BC subtype that lack the expression of estrogen receptor (ER), progesterone receptor (PR), and epidermal growth factor receptor 2 (HER2). Although accounting for 12–17% of all BC cases, TNBC are responsible for a significant proportion of BC-induced deaths due to their high rates of recurrence, metastatic spread, and limited therapeutic options [15]. In the present study, we aimed to better characterize the tumor-promoting actions of the IR-A vis a vis the IR-B in TNBC cells in response to insulin, and identify IR-A-specific gene profiles, which might reveal novel therapeutic approaches.

## 2. Materials and Methods

A detailed description of antibodies and reagents and protocols for the assessment of a wound healing migration assay, soft-agar colony formation, IR isoform mRNA expression, real-time PCR, mouse allografts, quantification of tumor-induced angiogenesis in zebrafish embryos, RNA extraction, library preparation, and sequencing and analysis of publicly available molecular datasets can be found in Supplementary Materials and Methods. 

### 2.1. Establishment of 4T1 Cells Overexpressing hIR-A or hIR-B Isoform

**Figure 1:**
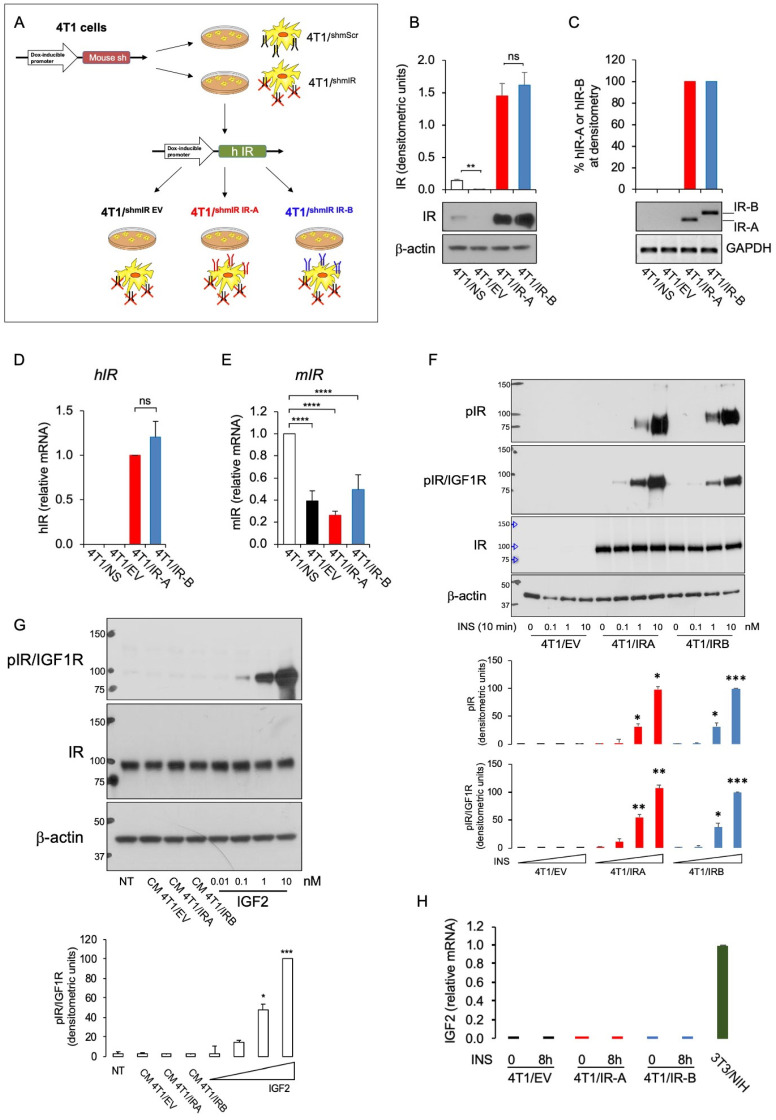
Characterization of 4T1 engineered cells. (**A**) Scheme depicting the strategy used to generate our cell models as described in Supplementary Methods. Briefly, 4T1 cells were infected with either a pTZ doxy-inducible lentiviral vector encoding a short hairpin RNA vector for murine IR (shmIR) or a scramble shRNA (shmScr, elsewhere referred to as 4T1/NS). 4T1shmIR cells were then exposed to a second infection with pTZ doxy-inducible lentiviral vector encoding for the human IR-A or IR-B or the corresponding empty vector to generate 4T1shmIR/hIR-A, 4T1shmIR/hIR-B, and 4T1shmIR/EV cells (referred as 4T1/IR-A, 4T/IR-B and 4T1/EV cells, respectively. (**B**) IR expression in 4T1/NS, 4T1/EV, 4T1/IR-A, or 4T1/IR-B cells was evaluated by Western blot analysis. Cells were grown in 10% FBS in the presence of doxycycline, then lysed, analyzed by SDS-PAGE and immunoblotted with the indicated primary antibodies to evaluate the expression of both mouse and human IR and β-actin. A representative blot of three independent experiments is shown. The graph on the upper panel represents the mean ± SE of densitometric analysis of three independent experiments, where hIR was normalized over β-actin. (**C**) IR isoform (IR-A and IR-B) transcripts were obtained from cell clones as indicated in (**B**). Products of PCR amplification were resolved on a 2.5% agarose gel, and images of PCR products from IR-B (Ex+11, 167 bp) and IR-A (Ex-11, 131 bp) obtained (middle panel). Graphical representation of PCR analysis indicates the percentage of IR-A mRNA calculated as follows: densitometric value of IR-A band/densitometric value of IR-A + IR-B bands (upper panel). Scanning densitometry was performed using ImageJ software. Results are expressed as means ± SE of three independent experiments. (**D**) Cells (as in **A**) were analyzed for hIR mRNA expression by qRT-PCR. Values are means ± SE of three separate experiments. (**E**) Cells were analyzed for mIR mRNA expression by qRT-PCR. 4T1/NS cells were used as control and GAPDH as housekeeping control gene. Values are means ± SE of three separate experiments. (**F**) 4T1/EV, 4T1/IR-A, and 4T1/IR-B cell monolayers unstimulated or stimulated with insulin at 0.1, 1.0, and 10 nM for 10 min were evaluated for total and phosphorylated IR proteins by Western blot using two different phosphoantibodies, as detailed in Methods. β-actin was used as loading control. The graph panels represent the mean ± SE of densitometric analysis of two independent experiments, where phosphorylated IRs were normalized over β-actin. (**G**) Cell conditioned medium from 4T1/EV, 4T1/IR-A, and 4T1/IR-B cells or IGF2 at the indicated doses were added to IR-A overexpressing mouse fibroblasts (R-/IR-A). A representative blot of two independent experiments is shown. The graph represents the mean ± SE of densitometric analysis of two independent experiments, where phosphorylated IRs were normalized over β-actin. (**H**) qRT-PCR measurement of IGF2 mRNA expression in 4T1/EV, 4T1/IR-A, and 4T1/IR-B cell monolayers stimulated or not with insulin 10 nM for 8 h. 3T3-NIH mouse fibroblasts were used as positive control. Values are means ± SE of two separate experiments. (ns, not significant; * *p* <0.05; ** *p* <0.01; *** *p* <0.001; and **** *p* <0.0001).

To compare cells expressing either the IR-A or IR-B isoforms, we established 4T1 cell clones characterized by conditional overexpression of either hIR-A or hIR-B and depletion of the endogenous *INSR*. 4T1 TNBC cells were obtained from American Type Culture Collection (ATCC, Manassas, VA, USA) and engineered within the first 6 months of purchase. We first infected 4T1 cells with mouse IR-specific, doxycycline-inducible short hairpin RNA (shmIR) or with non-silencing, scramble shRNA (Scr), to obtain 4T1shmIR and 4T1shmScr cells, respectively. We then infected 4T1shmIR cells with either an empty vector (EV) or with vectors containing cDNA for hIR-A or hIR-B, to obtain 4T1shmIR/EV, 4T1shmIR/hIR-A and 4T1shmIR/hIR-B, elsewhere referred to as 4T1/EV, 4T1/IR-A, and 4T1/IR-B cells, respectively (Figure 1A). Briefly, we transiently transfected human embryonic kidney HEK293T cells with the pTZ doxy-inducible lentiviral vector encoding for the short hairpin RNA vector for murine IR (shmIR) or with non-silencing (NS), scramble shRNA, using the Trans-Lentiviral Packaging System according to the manufacturer’s instructions. At 48 h post-transfection, we applied virus-containing medium to proliferating 4T1 cells, which were then trypsinized and re-plated in medium containing puromycin for selection. We then exposed 4T1shmIR cells to a second infection with pTZ doxy-inducible lentiviral vector encoding for either the human IR-A or IR-B or the corresponding empty vector to obtain 4T1shmIR/hIR-A, 4T1shmIR/hIR-B, and 4T1shmIR/EV cells (elsewhere referred to as 4T1/IR-A, 4T/hIR-B, and 4T1/EV cells, respectively). 4T1shmNS and 4T1shmIR/EV cells (elsewhere indicated as 4T1/NS and 4T1/EV) were used as control cells. We confirmed the silencing of the endogenous mIR and the expression of hIR-A and hIR-B isoforms in generated clones by Western blotting and qRT-PCR analysis. Cells expressing similar levels of hIR-A or hIR-B were chosen for further experiments. Cells were cultured for no more than five passages and were routinely screened for mycoplasma contamination using the VenorGeM OneStep Mycoplasma Detection Kit (CN 11-8025). 

### 2.2. Cell Proliferation and Viability Assay

The effect of insulin stimulation on cell proliferation was determined by cell counting. Briefly, 21,000 cells were seeded in a 12-well plate and allowed to adhere. One day later cells were serum-deprived for 24 h and then treated with 10 nM insulin every 24 h for two days. In the end, cells were detached and counted after Trypan blue exclusion.

Dose-response experiments for evaluating cell viability in cells exposed to insulin were conducted by the methyl thiazolyl tetrazolium (MTT) test (Amersham Biosciences, UK). Briefly, cells were seeded in 96-well plates. After 24 h, cells were grown in medium containing 0.1% BSA for a further 24 h and then exposed to insulin at concentrations of 0.1, 1, and 10 nM for 48 h. Cells were then incubated with medium containing 0.5 mg/mL MTT and processed according to the manufacturer’s instructions. 

### 2.3. IGF2 Measurement in Cell Conditioned Medium 

To prepare the conditioned medium (CM), 4T1 cell clones were seeded in 100 mm dishes in regular medium. After 24 h, cell monolayers were washed three times in PBS and then grown in medium containing 0.1% BSA. After a further 36 h, CM was collected and centrifuged at 1500 rpm for 5 min at 4 °C to remove cell debris.

#### 2.3.1. Biological Assay

To detect the presence of biologically active IGF2 in CM from 4T1 cell clones, cell monolayers of mouse fibroblasts lacking an IGF1 receptor and stably overexpressing IR-A (R-/IR-A cells) [8] were incubated with CM from 4T1 cell clones or with reference doses of IGF2, for 10 min. R-/IR-A cells were then solubilized and phosphorylated IR-A evaluated by Western blot analysis with the phospho-antibody anti-pIR/IGF1R (19H7, Santa Cruz Biotechnology, Santa Cruz, CA, USA).

#### 2.3.2. IGF2 mRNA Measurement

Total cellular RNA was extracted using TRIzol Reagent according to the manufacturer’s protocol. qRT-PCR was used to confirm the expression levels of mRNAs. Total RNA (2 μg) was reverse transcribed using the ThermoScript RT (Invitrogen, Carlsbad, CA, USA) and oligo (dT) primers. Synthesized cDNA was combined in a qRT-PCR reaction using the following primers: Fw GACCGCGGCTTCTACTTCAG; Rv AAGAACTTGCCCACGGGGTAT. The ΔΔCt method of relative quantification and SYBR Green chemistry were used to measure IGF2 mRNA. GAPDH was used as an endogenous control for normalization.

### 2.4. Cell Migration and Invasion Assay

To measure migration, cells were seeded in six-well plates to near confluency. Briefly, 24 h after seeding, cells were serum starved for a further 24 h, scratched using a sterile p20 tip (time 0 h) and allowed to migrate into the wound for 6–24 h in response to 0.1, 1, and 10 nM insulin. Pictures of the wound were taken at 0, 6, and 24 h using the X10 lens (Olympus, Tokyo, Japan). The wound areas were analyzed using the following formula: wound area (% of control) = (wound area after the indicated period/initial wound area) × 100.

Insulin-evoked cell invasion was assessed using transwell filters (8.0 μm) (BD Biosciences, Franklin Lakes, NJ). 4T1 BC cells (1 × 10^5^) overexpressing either hIR-A or hIR-B or control cells with silenced IR were seeded into the upper chamber containing a Matrigel-coated membrane. A serum-free medium (150 μL) was added to the upper chamber, and 0.1, 1, and 10 nM insulin (500 μL) was added to the lower chamber. After 18 h, cells on the top of the membranes were detached with a cotton swab and membranes stained with 0.05% crystal violet in phosphate-buffered saline (PBS) plus 20% ethanol. Thereafter, membranes were washed with water, and crystal violet from stained cells was solubilized with 10% acetic acid for 30 min and measured by reading absorbance at 595 nm. Cell invasion after insulin stimulation was calculated by using as basal control the number of cells migrated at the bottom side of the membranes in the absence of insulin.

### 2.5. Mouse Allografts

To study the effect of IR isoforms on tumor growth in immunocompromised animals, 4T1 BC cells overexpressing either hIR-A or hIR-B or IR-depleted control cells were injected orthotopically into female nude mice (nu/nu Swiss; Envigo Laboratories, Milan, Italy) and tumor growth was monitored. Briefly, 45-day-old athymic nude female mice were maintained in a sterile environment. At day 0, exponentially growing 4T1/EV, 4T1/IR-A, or 4T1/IR-B cells (1.0 × 10^4^ per mouse) were injected into the mammary fat pad region in 0.05 mL of Matrigel (BD Biosciences, Bedford, MA) (Matrigel/PBS ratio of 1:3). To evaluate the effect of chronic hyperinsulinemia, mice were divided into six groups, according to cell clone injection and treatments, and either saline or insulin Glargine was administered by subcutaneous (s.c.) injection 5 days/week for 25 days. More details are given in Supplementary Materials and Methods. Animal studies were performed in accordance with the principles of the Declaration of Helsinki and the Italian law D.L. 26/2014. They were carried out also in accordance with the Guide for the Care and Use of Laboratory Animals of the US National Institutes of Health (2011), and the Directive 2010/63/EU of the European Parliament. Animal care, euthanasia, and experiments were performed according to the principle of the 3Rs (replacement, reduction, and refinement) [16] and the institutional guidelines of the University of Calabria, Italy. The project was approved by the local ethical committee. 

### 2.6. Zebrafish Studies

Engineered 4T1 cells were initially cultured in RPMI serum, with addition of 10% FBS, glutamine, Pen Strep, and doxycycline (1.5 µM). At 72 h before the implantation, cells were exposed to serum deprivation. Starting from 48 h before the implantation, cells at approximately 50–80% confluence and during logarithmic growth were treated with insulin (10 nM). 

Zebrafish care and maintenance: Adult zebrafishes were maintained according to national (Italian D.lgs 26/2014) and European laws (2010/63/EU and 86/609/EEC) controlling experiments on live animals. Embryos, collected by natural spawning, were staged and raised at 28 °C in fish water (Instant Ocean, 0.1% methylene blue). Dechorionated embryos at 48 h post-fertilization (hpf) were anesthetized with 0.04 mg/mL of tricaine (Sigma-Aldrich). 

Procedure of tumor xenografts in zebrafish embryos: Tumor cells of each experimental group were stained with the red fluorescent viable dye CM-DiI (Invitrogen, Carlsbad, California, USA) and resuspended in PBS with doxycycline (1.5 µM) and insulin (10 nM). Afterward, tumor cells were implanted into the subepidermal space, close to the sub-intestinal vessels (SIV) plexus, of 48 h post-fertilization (hpf) Tg(fli1a:EGFP)y1 zebrafish embryos [17] using a microinjector FemtoJet (Eppendorf, Hamburg, Germany), equipped with a micromanipulator InjectMan NI 2 (Eppendorf, Hamburg, Germany). The injection of the cell suspension in the correct region of the embryo body is fundamental for obtaining a positive angiogenic response. For this purpose, we considered only embryos showing peridermal protrusion after cell injection and discarded embryos where cells were injected into the yolk. After the implantation, embryos were incubated at 32 °C, an intermediate temperature between the 28 °C most appropriate for zebrafish maintenance and 37 °C, best for mammalian cell growth and metabolism. At 24 h post-injection (hpi), each implanted embryo was imaged by epifluorescence microscopy (Leica M205FA; Wetzlar, Germany) equipped with a digital camera (gLeica DFC450C; Wetzlar, Germany), using the same acquisition parameters. Quantification of tumor-induced angiogenesis is described in detail in Supplementary Materials and Methods.

### 2.7. RNA-Seq Data Analysis

Total RNA extraction, library preparation, and sequencing are described in Supplementary Methods. Low-quality reads and sequencing adaptors were removed from the raw reads using Cutadapt [18]. Then, filtered reads were aligned on a reference indexed transcriptome (GENCODE GRCm38.p6 release M23) and quantified in a single step using Salmon [19]. The raw count matrix was then imported into Rstudio (R V 3.5.2) for statistical and differential expression analysis. Precisely, raw counts were normalized, and differentially expressed transcripts were identified using the LIMMA package (Bioconductor) [20]. We considered as differentially expressed all the transcripts with a Log2FC > 0.6 or < −0.6 and an adjusted *p*-value (Benjamini–Hochberg correction) < 0.05. Finally, we selected all the genes which were transcribed for the differentially expressed transcript in order to perform the MITHrIL pathway analysis [21]. Metabolic and signaling pathways were considered dysregulated if they had a corrected accumulator ≠ 0 and an adjusted *p*-value (Benjamini–Hochberg correction) < 0.05.

### 2.8. Survival Analyses of TCGA BRCA Samples

To perform a survival analysis of The Cancer Genome Atlas (TCGA) BC patients with high and low IR isoform expression, we retrieved the splice-junction expression matrix from Firebrowse (http://firebrowse.org/ accessed on 15 April 2021). Then, we gathered all clinical data of these BC patients from cBioPortal (https://www.cbioportal.org/ accessed on 15 April 2021). Precisely, raw counts associated with the splice-junction of exons 10–12 were used to quantify the IR-A isoform, while the minimum values of the raw counts related to the splice-junction of exons 10–11 and exons 11–12 were used for the quantification of the IR-B isoform. Once we quantified IR isoforms, the distribution of their expression values was computed across all samples to determine all possible cutoffs (from 5th to 95th percentile of the expression value). Therefore, for each percentile p, IR isoform expression > p was considered highly expressed. On the other hand, IR isoform expression < p was deemed to be lowly expressed. After that, we calculated the survival curves by using the R package Survival (https://cran.r-project.org/web/packages/survival/index.html accessed on 15 April 2021). The *p*-value obtained for each curve was evaluated by using a Cox proportional-hazards model. This process was iterated for each value of p (from 5th to 95th percentile). We chose the value associated with the lower *p*-value as the cutoff to discriminate patients according to their IR isoforms expression. This analysis was performed for each survival measurement available on TCGA, such as overall survival (OS), disease-specific survival (DSS), disease-free survival (DFS), and progression-free survival (PFS). Survival values were presented in terms of months. All survival curves were plotted by using the R package ggplot2 and ggpubr.

### 2.9. Statistical Analysis

We used the Student’s *t*-test for unpaired samples when comparing means in two groups. We used one-way ANOVA followed by post hoc analysis of significance (Bonferroni test) to calculate differences between means when comparing more than two groups. The level of significance was set at *p* < 0.05. Statistical analysis was conducted with GraphPad Prism6 (GraphPad Software, San Diego, CA, USA). Results were expressed as means ± SE.

## 3. Results

### 3.1. Establishment and Characterization of Cells Overexpressing hIR-A or hIR-B Isoform

To investigate the specific relevance of IR isoforms in BC, we used 4T1/IR-A and 4T1/IR-B cells, designed to overexpress the hIR-A or hIR-B isoform after doxycycline induction (Figure 1A). In the presence of doxycycline, 4T1/EV cells showed undetectable levels of IR protein as compared to control 4T1shmScr (4T1-NS) cells (Figure 1B), while 4T1/IR-A and 4T1/IR-B expressed similar levels of IR protein (Figure 1B). We confirmed by RT-PCR with isoform specific primers that only hIR-A or hIR-B mRNA was expressed in the selected engineered clones (Figure 1C). Moreover, using primers that specifically recognize common regions of the two isoforms, we confirmed by qRT-PCR that 4T1/IR-A and 4T1/IR-B cells expressed similar levels of either hIR-A or hIR-B mRNA (Figure 1D). No hIR mRNA was detected in 4T1/NS or 4T1/EV cells (Figure 1C,D). 4T1/EV, 4T1/IR-A and 4T1/IR-B cells showed effective depletion of endogenous mIR (Figure 1E). 

Dose-response curves of IR phosphorylation after insulin stimulation showed a similar pattern in 4T1/IR-A and 4T1/IR-B cells. In both cell lines, IR phosphorylation was undetectable in the absence of insulin (Figure 1F). As expected, no IR phosphorylation was observed in 4T1/EV cells either in the absence or in the presence of insulin stimulation. We then evaluated whether 4T1 cell clones could express autocrine IGF2. To rule out the possibility that a biologically significant amount of IGF2 protein could accumulate in cell conditioned medium (CM), we serum starved 4T1 cell clones, collected CM and measured IGF2 by biological assay. When added to mouse fibroblasts lacking IGF-1R and overexpressing IR-A (R-/IR-A cells), CM was unable to induce IR phosphorylation while a clear signal was obtained with the addition of exogenous IGF2 (starting from 0.1nM) **(**Figure 1G). In accordance with these findings, qRT-PCR showed undetectable levels of IGF2 mRNA (Figure 1H). Together, these results indicate that 4T1 cell clones do not express biologically significant concentrations of IGF2. 

### 3.2. IR-A Expression Is Associated with Enhanced Migration, Invasion, and Anchorage-Independent Growth 

**Figure 2:**
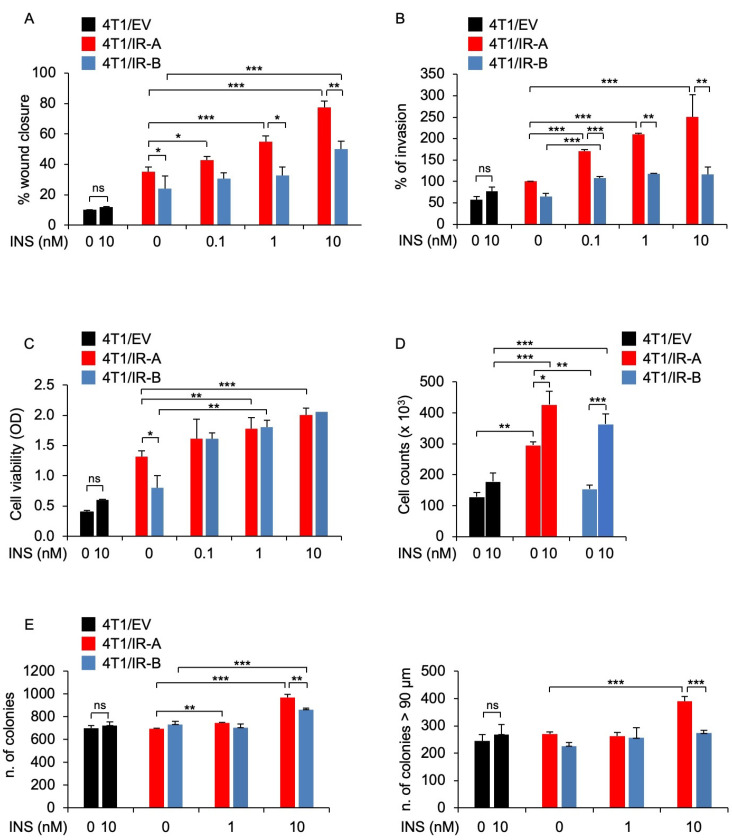
Biological responses of 4T1 engineered cells. (**A**) Wound healing assay. Cells were seeded onto 6-well plates till confluence. After 24 h, we generated wounds in the confluent monolayers. Incubation in serum-free medium was continued for additional 30 h in the presence or absence of 0.1, 1.0, and 10 nM insulin. Randomly chosen wound fields were photographed under a microscope at time = 0 and after 6 h, and 24 h. The histogram represents the mean of the migration index calculated as follows: wound area after the indicated period/initial wound area. Experiments were performed in triplicates and data calculated as means ± SE. Statistical significance was analyzed using the Student’s *t* test. (**B**) Cell invasion. Cells were seeded onto Matrigel-coated chambers in the presence or absence of insulin (0.1, 1.0, and 10 nM) for 18 h. Cells migrated to the lower compartment and adhering to the bottom surface of the membrane were quantified. The number of migrated cells after insulin exposure was expressed as the percentage of migrated cells over 4T1/IR-A cells migrated in the absence of insulin (basal). Data are presented as means ± SE of three independent experiments. (**C**) Cell viability. 4T1/EV, 4T1/IR-A, or 4T1/IR-B cells were incubated with or without insulin at doses of 0.1, 1.0, and 10 nM and evaluated by MTT assay. Values represent the mean ± SEM of three independent experiments performed in triplicate. (**D**) Cell proliferation. The same cells as in (**C**) were incubated with or without insulin (10 nM) and cell number measured by trypan blue exclusion assay. Values are means ± SE of three independent experiments. (**E**) Colony formation. Cells were seeded in soft agar, as described in Methods, and grown in 5% charcoal-stripped serum for 3 weeks. Colonies were then stimulated or not with insulin at the concentrations of 1.0 and 10 nM, stained with MTT, and photographed. The first histogram represents the number (mean ± SE) of total colonies from three independent experiments, each in duplicate wells. The second histogram represents the bigger colonies (mean ± SE) counted. (ns, not significant; * *p* < 0.05; ** *p* < 0.01; *** *p* < 0.001).

To assess whether hIR-A or hIR-B expression may differentially affect biological responses to insulin in 4T1 cells, we first evaluated the effects of insulin on migration and invasion of 4T1/IR-A and 4T1/IR-B cells. As shown in Figure 2A and in Figure S1, the ability of 4T1/IR-A cells to migrate in a wound healing assay, represented as the percentage of wound closure, was stimulated by insulin in a time- and dose-dependent manner and was significantly enhanced after 24 h as compared to 4T1/IR-B and 4T1/EV cells. Additionally, the ability to invade Matrigel-coated filters upon insulin exposure was also dose-dependent and strikingly greater in 4T1/IR-A cells as compared to 4T1/IR-B and 4T1/EV cells (Figure 2B). However, in 4T1/IR-A and 4T1/IR-B monolayer cell cultures, insulin showed similar stimulating effects on viability and growth, as evaluated by the MTT assay (Figure 2C) and cell number counts (Figure 2D). Instead, it was significantly more effective in stimulating anchorage-independent growth of 4T1/IR-A compared to 4T1/IR-B cells in both number and size of colonies (Figure 2E and Figure S2). Together, these results indicate that in 4T1/IR-A cells, insulin is considerably more effective in stimulating motility, invasion, and anchorage-independent growth, all critical hallmarks of tumor progression.

### 3.3. IR-A Enhances BC Growth and Metastasis In Vivo

**Figure 3:**
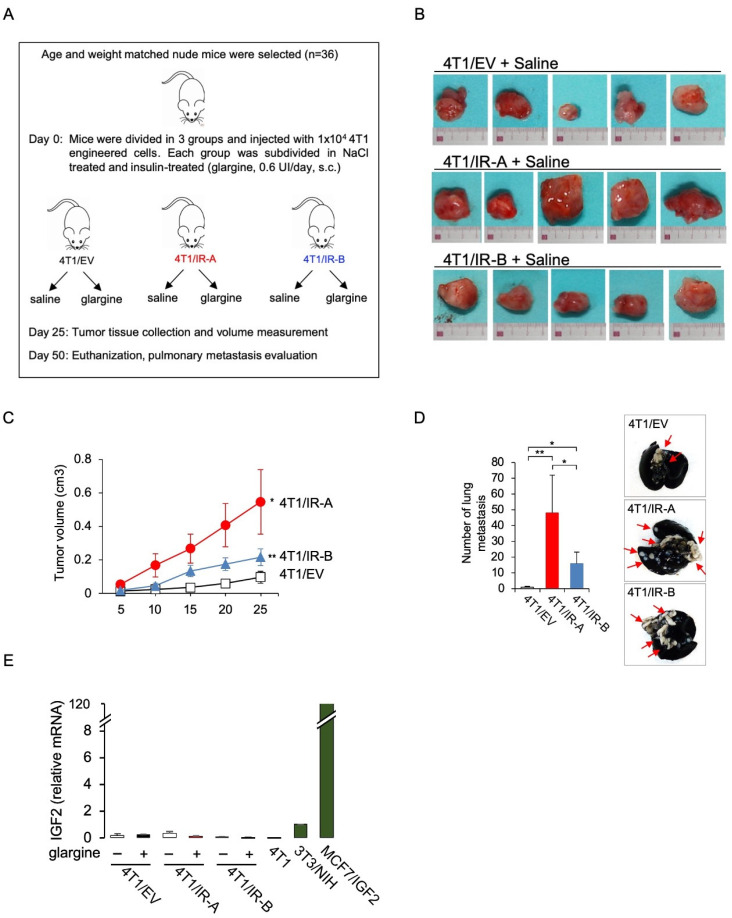
Tumor growth in immunocompromised mice. (**A**) Flowchart depicting the protocol scheme for the animal study. Female athymic nude mice were inoculated with 4T1 engineered cells. On the seventh day after inoculation 4T1/EV, 4T1/IR-A, or 4T1/IR-B cells were treated or not with 10 nM insulin glargine, given s.c. for 5 days/week (n = 6 for each group). At day 25, tumor volume was measured and tumor tissue was collected. Mice were sacrificed at day 50 and pulmonary metastasis evaluated. (**B**) Images of explanted tumors at day 25. Scale bar: 3 cm. (**C**) Graph showing the tumor volume (cm^3^) in 4T1/IR-A, 4T1/IR-B and in control (4T1/EV) inoculated-mice. The data are the mean ± SE of the values obtained in five animals per group. N.S., *p* > 0.05; * *p* < 0.05; and ** *p* < 0.01, by ordinary one-way ANOVA followed by post hoc analysis of significance (Bonferroni test) for the comparison between more than two groups. (**D**) Enumeration of lung metastases by in vivo examination in saline-treated mice inoculated with 4T1/EV, 4T1/IR-A, or 4T1/IR-B cells. The data are the mean ± SE of the values obtained in five animals per group (left panel). Representative images of India ink-filled lungs dissected from 4T1 tumor-bearing mice on day 50 (right panel). (**E**) qRT-PCR measurement of IGF2 mRNA in mice tumors. NIH-3T3 and MCF7/IGF2 cells were used as positive controls. Data are mean ± SE of two independent biological replicates. (ns, not significant; * *p* <0.05; ** *p*< 0.01).

We next examined whether the two hIR isoforms might differ in their ability to promote in vivo tumor formation using orthotopic mice allografts of the various 4T1-engineered cells. The 10^4^ cells were mixed with Matrigel/PBS and injected into the mammary fat pads of 8 weeks old female Nu/Nu mice. For each condition, we considered only doxycycline-treated groups supplemented or not with insulin at 0.6 units per day. Five mice/groups were analyzed. After 25 days, we explanted primary tumors and measured them (Figure 3A). Notably, tumor volumes derived from saline treated 4T1/IR-A-injected cells were significantly increased as compared to those derived from 4T1/IR-B cells or 4T1/EV cells (Figure 3B,C). Glargine-treated animals showed comparable results with no significant difference between saline-treated and glargine-treated mice (Figure S3). Mice weight did not show any significant difference among treatment groups. On day 50, mice were sacrificed, and distant metastases evaluated. All mice injected with 4T1/IR-A and 4T1/IR-B cells developed massive pulmonary metastases. However, metastatic nodules were significantly more numerous in mice injected with 4T1/IR-A cells than in mice injected with 4T1/IR-B or 4T1/EV cells (Figure 3D). Insulin glargine-treated mice showed similar results (not shown). Notably, tumors removed from mice expressed only trace amounts of IGF2 mRNA (Figure 3E). Taken together, these results indicate that cells expressing the hIR-A isoform have a greater ability to promote tumor growth and metastatic spread than cells expressing the hIR-B.

### 3.4. In Vivo Analysis of Tumor-Induced Angiogenesis

**Figure 4:**
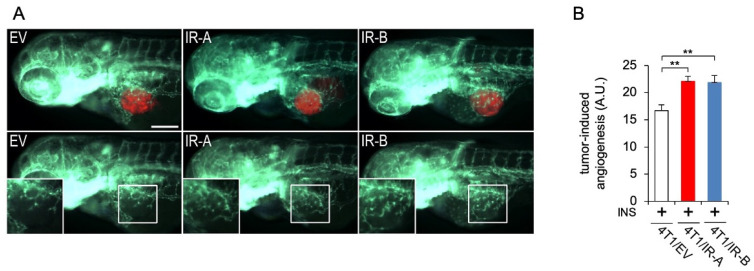
Tumor-induced angiogenesis assay in zebrafish embryos. (**A**) Representative images of 24 hpi Tg(fli1a:EGFP)y1 zebrafish larvae implanted with red fluorescence-stained 4T1/EV (EV), 4T1/IR-A (IR-A), and 4T1/IR-B(IR-B) cells. The red channel was omitted in the lower panels to highlight the differences in tumor-induced microvascular network between the experimental groups. Digital magnifications of graft regions (white box) are showed in the lower panels. All images are oriented so that rostral is to the left and dorsal is at the top. The same exposure time was used for all images. Scale bar: 100µm. (**B**) Histogram resulting from the quantification of areas corresponding to tumor-induced endothelial structures in 24 hpi embryos. The implantation of 4T1/IR-A and 4T1/IR-B similarly stimulated angiogenesis compared to control embryos (4T1/EV). Statistical analyses were carried out using one-way ANOVA followed by Tukey’s multiple comparison test: ** *p*< 0.01.

To analyze the proangiogenic potential of 4T1/EV (CTR), 4T1/IR-A, and 4T1/IR-B cell lines, we took advantage of tumor xenografts in zebrafish embryos. The embryo transparency associated with the availability of transgenic lines expressing fluorophores in endothelial lineages, such as the Tg(fli1a:EGFP)y1 line [22], represents a unique model to quickly visualize in vivo tumor-induced angiogenesis [23]. All three cell lines, pre-incubated with insulin and implanted in 48 hpf Tg(fli1a:EGFP)y1 embryos, induced an intricate network of endothelial sprouts deriving from SIV plexus and the CCV within 24 hpi (Figure 4A). In this experimental approach, both 4T1/IR-A and 4T1/IR-B were shown to be more potent than 4T1/EV control cells in stimulating tumor-induced angiogenesis in zebrafish embryos (Figure 4B).

### 3.5. Gene Expression Regulation by IR-A and IR-B

To provide further insight into the different ability of the IR-A and IR-B to modulate biological responses of TNBC cells, we performed RNA-seq on total RNA extracted from serum-starved 4T1/EV, 4T1/IR-A, and 4T1/IR-B cells incubated with 1.5 µM doxycycline and then stimulated with either vehicle or insulin (10 nM) for 3 h and 8 h. 

**Figure 5:**
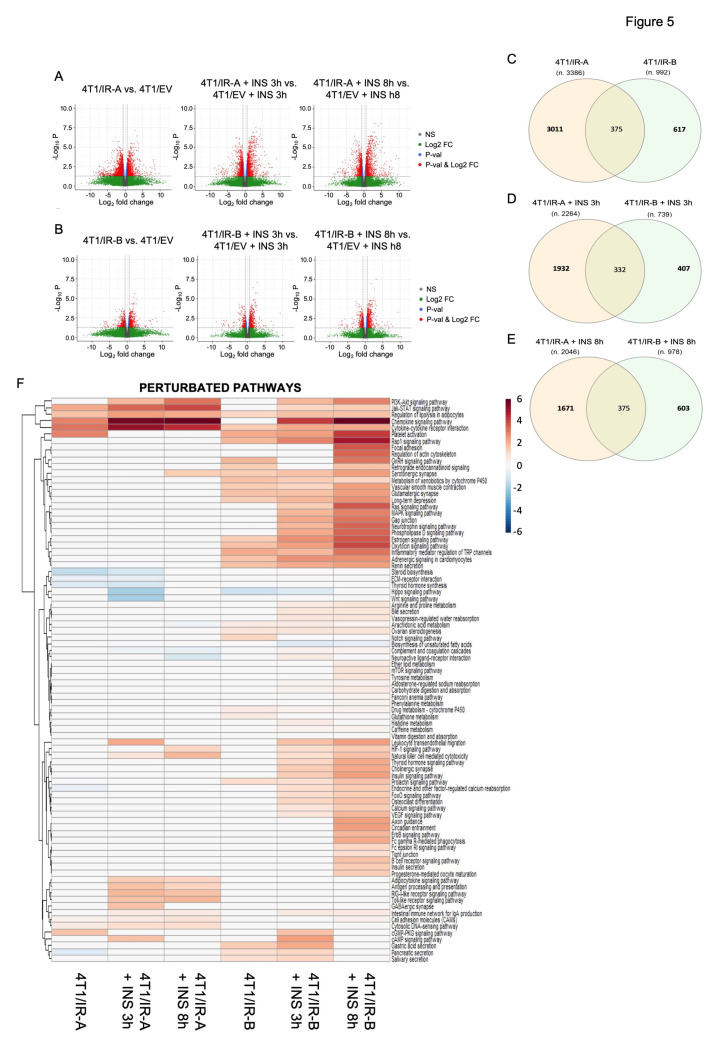
RNA-Seq transcriptome profiling. (**A**) Volcano plots show differentially expressed transcripts (in red) found with the following comparison IR-A vs. EV, IR-A_INS_3 h vs. EV_INS_3 h, and IR-A_INS_8 h vs. EV_INS_8 h. (**B**) Volcano plots show the differentially expressed transcripts (in red) with the following comparison IR-B vs. EV, IR-B_INS_3 h vs. EV_INS_3 h, and IR-B_INS_8 h vs. EV_INS_8 h. (**C**) Overlaps of the differentially expressed transcripts between 4T1/IR-A and 4T1/IR-B without insulin treatment. (**D**) Overlaps of the differentially expressed transcripts between 4T1/IR-A and 4T1/IR-B after insulin treatment for 3 h. (**E**) Overlaps of the differentially expressed transcripts between 4T1/IR-A and 4T1/IR-B after insulin treatment for 8 h. (**F**) Heatmap of the dysregulated pathways. This heatmap shows dysregulated pathways found by MITHrIL using the results of the RNA-Seq data analysis. Pathways were colored accordingly to their corrected accumulator values calculated by the MITHrIL algorithm. The red color means upregulation while the blue color means path downregulation. Pathways that were not found to be statistically dysregulated are colored in white.

We considered as differentially expressed all the transcripts with a Log2FC > 0.6 or <−0.6 and an adjusted *p*-value (Benjamini–Hochberg correction) < 0.05. The analysis revealed a considerable number of differentially expressed transcripts in both 4T1/IR-A and 4T1/IR-B-stimulated cells, suggesting important roles of IR isoforms in regulating gene expression (Figure 5A,B). Notably, 4T1/IR-A cells showed a slight predominance of upregulated genes in the stimulated condition (Figure 5A,B). Upon insulin exposure of 4T1/IR-A cells, 2264 (1993 genes) and 2046 (1811 genes) transcripts were differentially expressed at 3 h and 8 h, respectively, when compared to 4T1/EV cells at the same time point of insulin exposure (Figure 5A). On the other hand, in 4T1/IR-B cells, 739 (701 genes) and 978 (918 genes) transcripts were differentially expressed at 3 h and at 8 h respectively when compared to 4T1/EV cells at the same insulin exposure (Figure 5B). The complete list of regulated genes and transcripts can be found in Additional file 1. Notably, the overlap between the regulated transcripts in 4T1/IR-A and in 4T1/IR-B was 11.05% at 3 h and 12.4% at 8 h (Figure 5C–E). 

### 3.6. Pathway Analysis

The differentially expressed genes (DEGs) identified from the RNA-Seq data were subjected to pathway analysis using the MITHrIL algorithm [21]. The underlying pathway topologies, composed of genes and their directional interactions, were obtained from the Kyoto Encyclopedia of Genes and Genomes (KEGG) database [24]. According to the MITHrIL results, genes belonging to certain signaling pathways were strongly modulated in 4T1/IR-A but not in 4T1/IR-B cells (Additional file 2). Pathways upregulated only in 4T1/IR-A included: (1) adipocytokine signaling pathway; (2) antigen processing and presentation; (3) cell adhesion molecules; (4) cytosolic DNA-sensing pathway; (5) RIG-I-like receptor signaling pathway; (6) toll-like receptor signaling pathway. Other signaling pathways were instead downregulated in 4T1/IR-A cells: (1) complement and coagulation cascades; (2) ECM-receptor interaction; (3) endocrine and other factor-regulated calcium reabsorption; (4) steroid biosynthesis; (5) thyroid hormone synthesis. In contrast, some pathways were upregulated in 4T1/IR-B but not in 4T1/IR-A cells, including: (1) adrenergic signaling in cardiomyocytes; (2) aldosterone-regulated sodium reabsorption; (3) arachidonic acid metabolism; (4) arginine and proline metabolism; (5) calcium signaling pathway, (6) drug metabolism-cytochrome P450; (7) estrogen signaling pathway; (8) FoxO signaling pathway; (9) insulin signaling pathway; (10) metabolism of xenobiotics by cytochrome P450. Finally, other signaling pathways were upregulated by both IR isoforms, although IR-A-induced regulation was consistently more pronounced (Additional file 2). These pathways included: (1) chemokine signaling pathway; (2) cytokine-cytokine receptor interaction; (3) Jak-STAT signaling pathway; (4) natural killer cell-mediated cytotoxicity. Figure 5F shows the heat map of the statistically significant regulated pathways found by MITHrIL. The unprocessed MITHrIL results which contain both statistically and non-statistically regulated pathways are included in Additional file 3. It is not surprising that most metabolic pathways were regulated more specifically in cells overexpressing the IR-B. In agreement with previous studies [4], the IR-B is a more powerful regulator of glucose metabolism than the IR-A. Importantly, the IR-B regulated several metabolic pathways including amino acid metabolism and estrogen signaling pathways, which may also play crucial roles in cancer growth and progression. Genes related to angiogenesis and/or invasion and metastasis, such as *vegfa*, *serpine2*, *mmp13*, *mmp9*, *mmp3* (upregulated), and *Bmp7* (downregulated) were modulated by insulin in both cell lines although more markedly in 4T1/R-A cells, while others such as *pdgfra* and *ephb4* were significantly upregulated only in 4T1/IR-A cells (Additional files 1 and 2). However, the most striking finding was that the IR-A specifically regulated genes with a key role in the innate immune responses upon cytosolic DNA sensing and TLR signaling. These genes principally belong to the family of type I/II interferon (IFN) stimulated genes (ISGs) and participate in response to DAMPS and antiviral response (*ADAR, RIG-I like, MAD5/IFIH1*) (Figure 5F, Additional file 2). IFNAR1 (interferon alpha and beta receptor subunit 1) was significantly induced only in IR-A overexpressing cells (Additional file 1). However, both IR isoforms upregulated genes implicated in cytokine–cytokine receptor interaction, chemokine signaling, the JAK-STAT signaling pathway, as well as natural killer cell-mediated cytotoxicity (Figure 5F, Additional file 2). Finally, both IR isoforms significantly upregulated *Notch4* and *Id1*, two genes implicated in the maintenance of stem-like phenotype in BC [25,26].

### 3.7. Validation of RNA Seq Analysis

**Figure 6:**
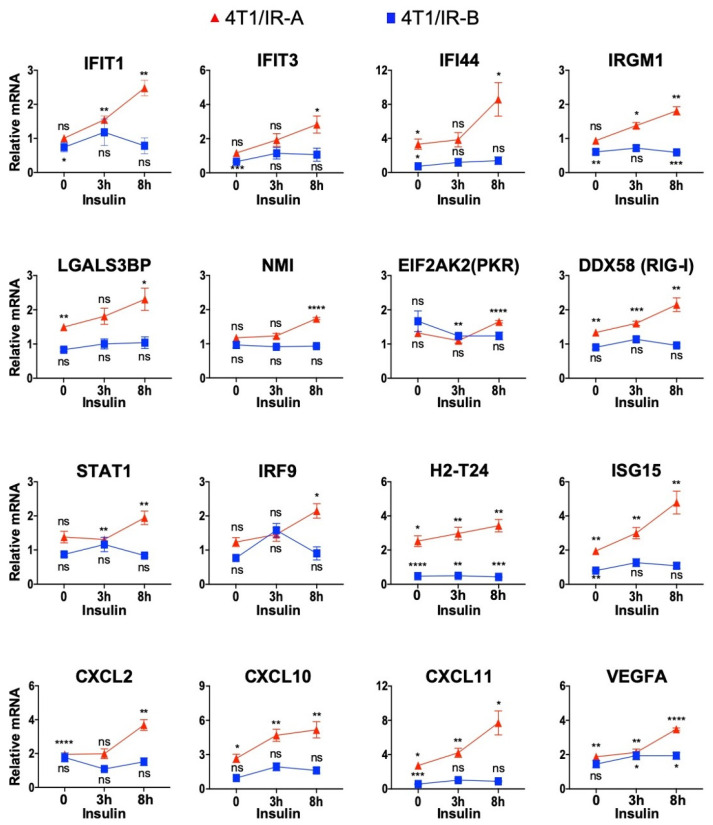
RNA-Seq transcriptome profiling validation through qRT-PCR analysis of selected genes. Data were normalized over the corresponding values obtained from control cells and are expressed as means ± SE from three different experiments. (ns, not significant; *p* > 0.05; * *p* < 0.05; ** *p* < 0.01; *** *p* < 0.001; and **** *p* < 0.0001).

To validate the results obtained with RNA sequencing, we measured the expression of a panel of 16 genes by qRT-PCR in 4T1/IR-A and 4T1/IR-B cells stimulated or not with insulin (Figure 6). Eleven of these genes, belonging either to the interferon, antiviral response, or the cytosolic DNA-sensing pathways, were chosen to validate their preferential activation in 4T1/IR-A cells. These genes included: *IFIT1*, *IFIT3* [27], *IFI44* [28], *IRGM1* [29], *NMI* [30], *ISG15* [31], *LGALS3BP* [32], *IRF9* [33], *STAT1* [34], *EIF2AK2/PKR* [35], *DDX58/RIG-I* [35]. Six of these 11 genes have been previously described as part of the so-called IFN-related DNA-damage resistance signature (IRDS), which has been associated with resistance to chemo- and radiotherapy (see Discussion). Three genes, *Cxcl2* [36], *Cxcl10* [37], and *Cxcl11* [38], belonging to the chemokine pathway, and *Vegfa* [39], a critical angiogenic factor, were also preferentially modulated in 4T1/IR-A cells (Additional file 1). Overall, the results from qRT-PCR (Figure 6) closely resembled the data obtained by RNAseq analysis (Additional file 1), confirming the differential modulation of gene expression by insulin in 4T1/IR-A and in 4T1/IR-B cells. 

### 3.8. Survival Analyses of Publicly Available Molecular Datasets

**Figure 7:**
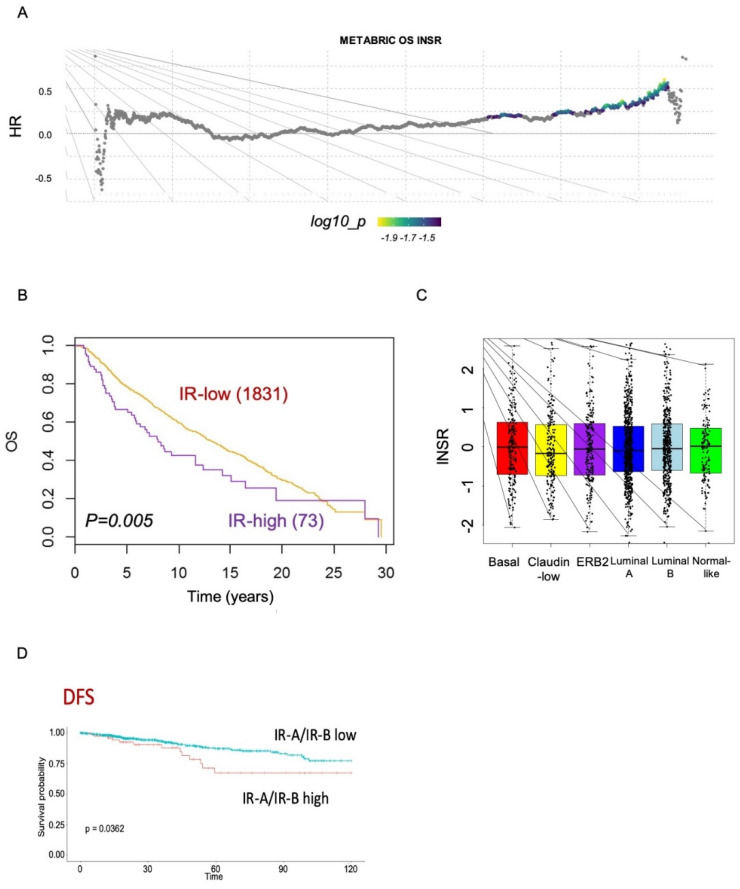
Survival analysis from METABRIC datasets. (**A**) Comprehensive survival analysis using suvivALL R package in the METABRIC datasets (1904 BC patients). Hazard ratios indicate the direction and magnitude of the association, and the colors show significance (bright colors indicate *p* < 0.05). (**B**) Overall survival (OS) in BC patients with either low or high IR tumor levels in the METABRIC cohort. (**C**) Box plots showing the distribution of IR expression levels in the different BC subtypes according to the METABRIC datasets. (**D**) DFS (disease-free survival) in BC patients from the TCGA dataset according to the IR-A/IR-B ratio.

**Figure 8:**
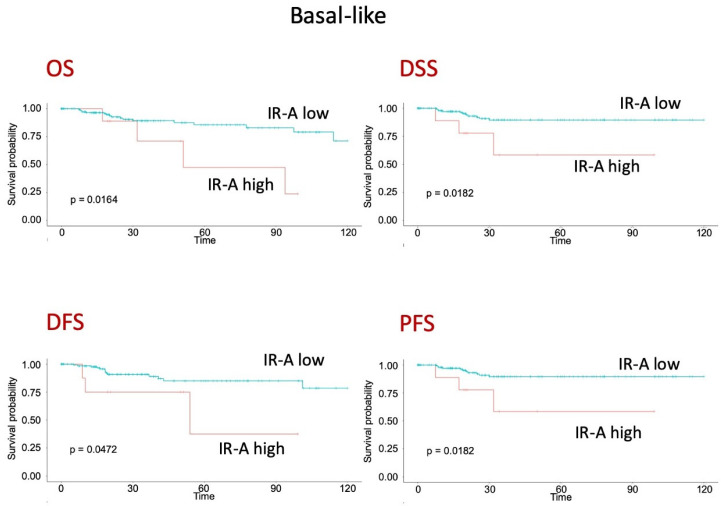
Survival analysis of patients with BC, basal-like molecular subtype, from TCGA dataset. OS (overall survival), DSS (disease-specific survival), DFS (disease-free survival) and PSF (progression-free survival) according to high or low IR-A expression.

To gain further insight on the prognostic value of IR and IR isoforms, we analyzed publicly available METABRIC and TCGA datasets, which are derived from Affymetrix microarray analysis and transcriptomic data, respectively. Both datasets provide expression data on the IR gene, whereas only TCGA provides data on IR isoform transcripts. The survival analysis of the METABRIC dataset showed that high IR expression was associated with worse OS, independently of the molecular subtype (Figure 7A–C). The TCGA dataset showed instead that a higher IR-A/IR-B ratio was clearly associated with worse DFS (Figure 7D). Significantly, in patients with the basal-like molecular subtype of BC, comprising most TNBCs [40], high IR-A expression was associated with worse OS, DSS, DFS, and PSF (Figure 8). 

## 4. Discussion

Our study was designed to analyze the biological responses elicited by insulin in TNBC cells and assess the specific contribution of the two different IR isoforms, the IR-A and IR-B. Thus, we established a tumorigenic TNBC murine cell model where the endogenous IR was silenced by an inducible shRNA approach and IR expression was reconstituted by transfecting either the human IR-A or IR-B. In vitro studies showed that both IR isoforms enhanced TNBC cell biological responses to physiologic doses of insulin. Notably, after insulin stimulation, IR-A overexpressing cells showed significantly stronger ability to migrate and invade through Matrigel than IR-B overexpressing cells, while both cell lines responded similarly to insulin in monolayer growth. IR-A-overexpressing cells additionally exhibited increased anchorage-independent growth, which was evident in both colony numbers and size when compared to IR-B-overexpressing cells. When transplanted into athymic mice, both IR-A- and IR-B- overexpressing TNBC cells developed tumors and distant metastases more rapidly than EV control cells, both after saline and insulin treatment. Significantly, cells overexpressing the IR-A formed significantly larger tumors and more massive metastases compared to cells overexpressing the IR-B. We did not observe a clear tumor promoting effect by insulin glargine administration, possibly indicating that endogenous mouse insulin already provided maximum tumor stimulation. We cannot exclude that IGF2, the second IR-A ligand, might contribute to the faster tumor growth and metastasis in animals inoculated with 4T1/IR-A cells when compared to 4T1/IR-B cells. However, we consider this possibility highly unlikely for the following reasons: (a) cultured 4T1 cells did not produce IGF2 and explanted tumor specimens contained only trace amounts of IGF2 mRNA; (b) IGF2 binds to IR-A with a 7–10 lower binding affinity respect to insulin, and, therefore, can displace insulin binding only when present at molar excess; (c) especially in insulin glargine-treated mice, the tumor IR-A is expected to be saturated by insulin that cannot be displaced by the lower affinity ligand, IGF2 [4]. In the zebrafish model, both IR isoforms significantly enhanced in vivo tumor angiogenesis compared to control cells, indicating that tumor-induced angiogenesis might play a significant role in IR-driven tumor growth. We cannot exclude that the sensitivity of the method was not high enough to detect small but biologically significant differences. More studies are needed to confirm the present evidence that both IR isoforms are equally effective in stimulating tumor angiogenesis. 

Taken together, these data indicate that in the presence of insulin, the IR-A and to a lesser extent the IR-B isoform elicit several biological responses from TNBC cells, which may explain the negative prognostic effect of hyperinsulinemia in obese patients with BC [14].

To our knowledge, these findings are very novel and in agreement with previous studies indicating that insulin might favor tumor growth and progression through its cognate receptor [41] in non-obese mice [42].

To provide a molecular basis for the differences in insulin-mediated biological responses elicited specifically by the two IR isoforms, we analyzed insulin-dependent whole transcriptome in TNBC cells overexpressing either the IR-A or IR-B human isoforms and validated this analysis by assessing a panel of genes by real-time RT-PCR. Whole transcriptomic analysis revealed that the regulation of defined signaling pathways was considerably different in cells expressing the IR-A compared to the IR-B. As expected, the IR-B showed a prevalent role in regulating genes implicated in metabolic pathways. These pathways, involving amino acid metabolism, arachidonic acid metabolism, drug metabolism-cytochrome P450, and FoxO signaling might also have an important but unappreciated role in regulating TNBC tumorigenesis and progression [4]. 

However, the different modulation of gene expression might explain the more potent effect of the IR-A in cancer progression. Genes related to angiogenesis, including *vegfa*, *pdgfra*, and *serpine2*, were induced by insulin in both 4T1/IR-A and 4T1/IR-B cells, although more markedly in 4T1/IR-A cells. Of note, the *serine protease serpine2* was one of the most significantly upregulated genes in 4T1/IR-A cells while only slightly upregulated in 4T1/IR-B cells. The serpine2 protein has been implicated in BC metastatic process through its ability to promote neo-angiogenesis and vascular mimicry, to act as an anticoagulant [43], to affect extracellular matrix remodeling, and polarization of tumor-associated macrophages [44]. Transcripts encoding for metalloproteinases, typical drivers of cancer invasion, metastasis, and angiogenesis [45], also followed a similar pattern of response to insulin stimulation. For instance, expression of metalloproteinase 13 (mmp13) was increased by more than 4-fold in 4T1/IR-A cells but only by approximately 1.3-fold in 4T1/IR-B cells. 

Moreover, the transcript for Bmp7 (Bone morphogenetic protein 7) was markedly downregulated in 4T1/IR-A cells but only slightly downregulated in 4T1/IR-B cells. Bmp7 is a matrix protease that breaks down collagen type IV and exerts pleiotropic and context-dependent biological effects. In BC model systems, downregulation of Bmp7 contributes to EMT, cell migration, and metastatic spread [46]. Moreover, the transcript for EphB4, an Eph/Ephrin receptor, was significantly upregulated only in 4T1/IR-A cells. EphB4 protein is aberrantly expressed in a variety of malignancies where it contributes to angiogenesis, invasion, and metastasis [47]. Consistent with the present data, we previously showed that EphB4 functionally interacts with the IR-A [48]. 

Remarkable is the finding of the unique action of the IR-A in regulating key pathways of innate immunity such as pattern recognition receptors (PRRs), e.g., RIG-I-like receptors and toll-like receptors (TLRs), and cytosolic DNA sensors. These pathways are all involved in cell defense by binding to exogenous pathogen-associated molecular patterns (PAMPs) and danger-associated molecular patterns (DAMPs) released by cells undergoing damage. PRRs ligands, such as various S100 proteins and HMGB1, were also upregulated. Normally, PPR activation by various PAMPs and/or DAMPs activates IRF3 and IRF6 transcription factors, which regulate the expression of IFN-I and in turn elicits the transcription of ISGs involved in immune activation and cell defense. In TNBC cells overexpressing the IR-A, insulin-induced upregulation of several ISGs resembles the previously described set of ISGs involved in the so-called IRDS (IFN-related DNA-damage resistance signature) associated with resistance to chemo- and radiotherapy [49]. Although the complex network of ISGs induced in IR-A-overexpressing TNBC cells might have dual and cell context-dependent effects on cancer hallmarks, it is generally acknowledged that a chronic IRDS-like signature has a tumor-promoting activity [49]. Among these genes, ISG15 has been involved in TNBC brain metastases and poor BC prognosis [31], and the IFN-β-STAT1-ISG15 signaling axis has been recognized as an oncogenic pathway in TNBC [50]. 

Insulin stimulation of both IR-A and IR-B-overexpressing cells upregulated other pathways linked to immunity, such as the chemokine signaling pathway, cytokine-cytokine receptor interaction, the JAK-STAT signaling pathway, and natural killer cell-mediated cytotoxicity, suggesting that both IR isoforms might share a role in immune regulation. However, our results suggest that the impact of the IR-A in the regulation of the immunity-related pathway is stronger and wider than the one mediated by the IR-B, as in fact two other immune-related pathways, the adipocytokine signaling pathway and antigen processing and presentation, were regulated by insulin solely in IR-A overexpressing cells.

Based on these findings, we can hypothesize that, in hyper-insulinemic patients, the overexpression of the IR-A in TNBCs may increase tumor progression through multiple mechanisms including the stimulation of gene expression programs associated with cell migration and invasion, EMT, and the stem-like phenotype as well as angiogenic and vascular mimicry. In addition, hyperinsulinemia may promote immune-evasion and tumor resistance to chemo and radiotherapy and immune checkpoint-based therapies through the stimulation of an IRDS-like signature as well as the production of cytokines and chemokines. By analyzing the TCGA dataset, we provided evidence that IR-A overexpression has a negative impact on OS and DFS in patients with a basal-like subtype, which includes most TNBCs. 

We certainly acknowledge that our study has some limitations. We did not compare the cellular response of insulin and IGF2, the other high-affinity ligand of the IR-A. However, this issue was addressed in previous studies using a different model system [8,48,51]. Nonetheless, to the best of our knowledge, the present study is the first to compare the in vitro and in vivo characteristics of IR-A and IR-B overexpressing TNBC cells and to report the whole transcriptomic analysis in response to insulin in these cells. These results highlighted substantial signaling differences between the two IR isoforms that might help in understanding their complex roles in both physiology and disease [4].

## 5. Conclusions

In conclusion, we found that IR-A overexpression in TNBC cells enhances in vitro and in vivo oncogenic features. While the IR-A was more potent than the IR-B in modulating features of tumor progression, the IR-B still contributed to regulate cancer cell growth and cancer-related angiogenesis. Moreover, we discovered that the insulin-dependent transcriptome in TNBC cells is largely dependent on the expressed IR isoform with only partial overlap. In particular, IR-A overexpression was particularly associated with gene expression programs involved in tumor progression and immune evasion, and resistance to cancer therapies. Analysis of METABRIC and TCGA datasets confirmed the complex role of IR isoforms in human BC. 

Collectively, these data can contribute to further understanding the negative prognostic effect of hyperinsulinemia in BC patients and can help identify novel molecular therapeutic targets. 

## Data Availability

Figures and datasets supporting the conclusions of this article are included in the article as additional files (Figures S1–S3, additional files 1–3). Any other data presented in this study are available on request from the corresponding author.

## Supplementary Materials

The following are available online at https://www.mdpi.com/article/10.3390/cells10113145/s1, Figure S1: Cell migration evaluated by wound-healing assay. 4T1/EV, 4T1/IR-A and 4T1/IR-B were treated with or without 10 nM of insulin. Black lines indicate the wound borders at the beginning of the assay and recorded 24 h post-scratching; Figure S2. Colony formation. Cells were seeded in soft agar and grown in 5% charcoal-stripped serum for 3 weeks and then treated or not with 10 nM of insulin. Colonies were stained with MTT and then photographed. Figure S3. Tumor growth in nude mice. Gross appearance of tumors obtained from 4T1/IR-A, 4T1/IR-B cells, compared to 4T1/EV inoculated mice, treated or not with insulin glargine.
