# Supplementary material for: Novel Mechanisms of Tumor Promotion by the Insulin Receptor Isoform A in Triple-Negative Breast Cancer Cells
Source: Cells. 2021 Nov 12;10(11):3145. doi: 10.3390/cells10113145 (PMC8621444; doi:10.3390/cells10113145)
Supplement: Supplementary file 1 [file cells-10-03145-s001.zip › cells-1337110-supplementary.pdf]

Supplementary Materials and Methods

Antibodies and reagents

Bovine serum albumin (BSA) and fibronectin were from Sigma-Aldrich (Saint Louis Missouri, USA); fetal calf serum (FCS), TRIzol Reagent, ThermoScript RT kit, SYBR Green MasterMix from Life Technologies, Inc. Laboratories (Paisley, UK); MTT, nitrocellulose membranes, HRP-conjugated secondary antibodies were from Amersham Biosciences (Little Chalfont, UK). The following antibodies were used: anti-IR (C-19, sc-711) (Santa Cruz Biotechnology, Santa Cruz, CA, USA), anti-phospho(p)IGF1R (Tyr1135/1136)/pIR (Tyr1150/1151) (19H7) (Cell Signaling Technology Inc., Beverly, MA, USA). Human insulin was purchased from Peprotech (Rocky Hill, NJ), insulin glargine (rDNA origin) was from Sanofi Aventis (Milano, IT).

Wound healing migration assay

To measure migration, cells were seeded in six-well plates to near confluency. After 24 h cell monolayers were scratched using a sterile p20 tip (time 0). Cells were allowed to migrate into the wound for 30 h. Pictures of the wound were taken at 0, 6, 24, and 30 h using x10 magnification. The wound closure area (% of control) was calculated as follows: wound area after the indicated period/initial wound area [1].

Soft-agar colony formation

Anchorage-independent growth was assessed as previously described. Briefly, a mixture of 0.66% agar and medium containing 5% CS-FCS was plated on the bottom of each well plate (hard agar). Then, cells suspended in a 5% CS-FCS medium containing 0.33% agar (soft agar) were plated on the top of the hard-agar layer. Top agar was then covered with culture media. Cells were cultured for 10 days in the presence or absence of ligands, as indicated. Colonies were visualized with 7 mg/mL methyl thiazolyl tetrazolium (MTT), photographed, and analyzed with NIH ImageJ.

IR isoform mRNA expression

mRNA expression of IR isoforms was measured by RT-PCR analysis (Bioline PCR Kit) using primers for flanking exons 10 and 12 and resolved on 2.5% agarose gel. The 167-bp and 131-bp DNA fragments, representing Ex11+ (IR-B isoform) and Ex11− (IR-A isoform), respectively, were quantified by densitometry analysis using NIH ImageJ. The proportion of IR-A expression levels was calculated as the densitometric value of band IR-A/densitometric values of bands IR-A + IR-B.

Quantitative real-time PCR

Total cellular RNA was extracted using TRIzol Reagent according to the manufacturer's protocol. qRT-PCR was used to confirm expression levels of mRNAs. Total RNA (2μg) was reverse transcribed using the ThermoScript RT (Invitrogen) and oligo (dT) primers. Synthesized cDNA was combined in a qRT-PCR reaction using primers for the gene of interest (Table 1). Real-time PCR was performed with an ABI 7500 Real-Time PCR System (Applied Biosystems) using probes, primer sets, and SYBR Green chemistry. Human GAPDH and β-actin were used for normalization in SYBR Green chemistry. mRNA quantification was performed using the comparative CT method (ΔΔCt).

Table S1.

mCXCL2	*Fw 5′-GCTGTCCCTCAACGGAAGAA-3′*<br>*Rv 5′-CAGGTACGATCCAGGCTTCC-3′*
mCXCL10	*Fw 5′-CAAGTGCTGCCGTCATTTTC-3′*<br>*Rv 5′-AATGATCTCAACACGTGGGC-3′*
mCXCL11	*Fw 5′-TGAGATGAACAGGAAGGTCAC-3′*<br>*Rv 5′-CAACTTTGTCGCAGCCGTTA-3′*
mDDX58	*Fw 5′-ACTTCGAACACGTTTAAATGCA-3′*<br>*Rv 5′-ATCCACTGTTCATATTTCTGGGT-3′*
mEIF2AK2	*Fw 5′-CGGGAAAACGAAACAGAAGAG-3′*<br>*Rv 5′-GAAACCTGGGGTATCACTGGC-3′*
mGAPDH	*Fw 5′-TGACGTGCCGCCTGGAGAAA-3′*<br>*Rv 5′-AGTGTAGCCCAAGATGCCCTTCAG-3′*
mH2-T24	*Fw 5′-CCTTCATCCACTACGACAGC-3′*

	Rv 5′-CGTATGTGAACTGGAGGGTG-3′
mIFI44	*Fw 5′-TTACACACGTGGATAGCCTG-3′*<br>*Rv 5′-AGGCAAAACCAAAGACTCCA-3′*
mIFIT1	*Fw 5′-CCAAGTGTTCCAATGCTCCT-3′*<br>*Rv 5′-GGATGGAATTGCCTGCTAGA-3′*
mIFIT3	*Fw 5′-CTGAACTGCTCAGCCCACA-3′*<br>*Rv 5′-TTCCCGGTTGACCTCACTCA-3′*
hIR isoform	*Fw 5′-CCAAAGACAGACTCTCAGAT-3′*<br>*Rv 5′-AACATCGCCAAGGGACCTGC-3′*
hIR total	*Fw 5′-CGTGGAGGATAATTACATCGTGTT-3′*<br>*Rv 5′-TGGTCGGGCAAACTTTCTG-3′*
mIR	*Fw 5′-CTACAGTGTTCGAGTCCGGG-3′*<br>*Rv 5′-TGGCAATATTTGATGGGACATCT-3′*
mIRF9	*Fw 5′-TGAAACTTAGGGTGGGGACT-3′*<br>*Rv 5′-CCCTCTTTGCTAGAGGGGTA-3′*
mIRGM1	*Fw 5′-ACGTTCCAGGAAGGCCACTA-3′*<br>*Rv 5′-AATACTCCTCAAACCCTGATCCA-3′*
mISG15	*Fw 5′-CGTGACTAACTCCATGACGG-3′*<br>*Rv 5′-GCAGTTCTGTACCACTAGCA-3′*
mLGALS3BP	*Fw 5′-GCCTGTCGTCAGAGACTTCC-3′*<br>*Rv 5′-TGCTGACCTCGATTCTTCGG-3′*
mNMI	*Fw 5′-GCCAGGTTAGTGTTTTCGAGG-3′*<br>*Rv 5′-CTACAGAACTCAGCACCCGC-3′*
mSTAT1	*Fw 5′-GTCATCCCGCAGAGAGAACG-3′*<br>*Rv 5′-GAGCAGAGCTGAAACGACCTA-3′*
mVEGFA	*Fw 5′-CTATTCAGCGGACTCACCAG-3′*<br>*Rv 5′-GGGAGTGAAGAACCAACCTC-3′*

Mouse allografts

The first group of mice (n = 6), injected with 4T1/EV cells was treated with 100 µl of vehicle (NaCl); the second group of mice (n = 6), injected with 4T1/EV cells was treated with 100 µl of glargine (0.6 U/die); the third group of mice (n = 6), injected with 4T1/IR-A cells was treated with 100 µl of

vehicle (NaCl); the fourth group of mice (n = 6), injected with 4T1/IR-A cells was treated with 100 µl of glargine (0.6 U/die); the fifth group of mice (n = 6), injected with 4T1/IR-B cells was treated with 100 µl of vehicle (NaCl); finally, the sixth group of mice (n = 6), injected with 4T1/IR-B cells was treated with 100µl of glargine (0.6 U/die). Doxycycline hyclate (Sigma) 2g/L, was delivered to the mice through drinking water, tap water + 3.0% sucrose (Sigma), in dark stained bottles and renewed every 3 days for 25 days. Allograft tumor growth was monitored twice a week by caliper measurements, along two orthogonal axes: length (L) and width (W). Tumor volumes (in cm^3) were estimated as described [2]. Mice weight was also monitored. On day 25, the primary tumors were explanted following a standard protocol. Specimens of tumors were frozen in nitrogen and stored at – 80°C, the remaining tumor tissues of each sample were fixed in 4% paraformaldehyde and embedded in paraffin for the histologic analyses. Mice were monitored for additional 25 days to allow the development of distant metastases and sacrificed on day 50. The organs of interest were then harvested for quantification of metastases to distant sites. Lung metastases were assessed by ex vivo examination. Briefly, murine lungs were filled with 15% India ink via the upper trachea and fixed in Fekete's solution (100 mL of 70% alcohol, 10 mL of 4% formalin, and 5 mL glacial acetic acid) overnight. White tumor nodules against a dark lung background were photographed. Metastatic lesions on the black lung surface were counted.

Quantification of tumor-induced angiogenesis in zebrafish embryos

The vascular anatomy of the developing zebrafish embryo has been described in detail and has a high structural homology to other vertebrates [3]. The use of vascular specific transgenic fluorophore expression allows for the detection of individual growing cells or cellular compartments and therefore the analysis of vessel formation [3]. We quantified tumor-induced angiogenesis using Fiji software. As an arbitrary unit (A.U.), we measured the EGFP area corresponding to endothelial structures that sprouted from the plexus of SIV and the common cardinal vein (CCV) in each imaged embryo. To this purpose, we selected a region of interest (ROI) in the area surrounding the graft of each embryo, in which only tumor-induced endothelial structures were included and normal developmental vessels were not considered. Afterward, we set the same threshold for the EGPF channel in each embryo, in order to exclude the background signal. Thus, we limited to the threshold the calculation of the area in

each selected ROI. The area from each experimental group was reported as mean ± S.E.M. Experiments were performed 3 times, considering at least 20 embryos in each experimental group. GraphPad Prism 5.0 (GraphPad Software, San Diego, CA) was used for statistical analysis. Statistical differences were evaluated using one-way ANOVA followed by Tukey's multiple comparison test.

Total RNA extraction, library preparation, and sequencing

The 4T1 cells were incubated overnight in medium without serum, then treated with insulin (10 nM) or not (control condition) for 3 and 8 hours (two biological replicates per condition, pooled twice). The timing of insulin stimulation was chosen on the basis of previous work [4,5]. Total RNA was extracted using a Total RNA purification kit (Norgen Biotek Corporation, Canada), following the manufacturer's instructions; its yield, 260/280, and 260/230 ratios were measured using a NanoDrop spectrophotometer (ThermoFisher Scientific, Inc., Waltham, MA, USA) and sent to IGA Technology Services for the subsequent processing. A TruSeq Stranded mRNA kit (Illumina, San Diego, CA) was used for library preparation following the manufacturer's instructions (library type: fr-firstrand). RNA samples were quantified, and quality tested by Agilent 2100 Bioanalyzer RNA assay (Agilent technologies, Santa Clara, CA) or Caliper (PerkinElmer, Waltham, MA). Final libraries were checked with both Qubit 2.0 Fluorometer (Invitrogen, Carlsbad, CA), and Agilent Bioanalyzer DNA assay or Caliper (PerkinElmer, Waltham, MA). Libraries were then prepared for sequencing and sequenced on paired-end 150 bp mode on NovaSeq6000 (Illumina, San Diego, CA).

Analysis of BC patients in METABRIC molecular dataset

We analyzed the publicly available METABRIC dataset (22522925) containing clinical information and microarray gene expression data (Log2 transformed intensity values) of a cohort of 2509 patients retrieved from cBioPortal for Cancer Genomics (http://www.cbioportal.org/). *INSR* expression was analyzed according to the BC molecular subtype and comprehensive survival analysis performed using the suvivALL R package [6]. Gene expression and clinical information were also filtered for missing values. The final filtering resulted in 1904 BC patients.

Supplementary references

1. Rigiracciolo, D.C.; Santolla, M.F.; Lappano, R.; Vivacqua, A.; Cirillo, F.; Galli, G.R.; Talia, M.; Muglia, L.; Pellegrino, M.; Nohata, N.; et al. Focal Adhesion Kinase (FAK) Activation by Estrogens Involves GPER in Triple-Negative Breast Cancer Cells. *J. Exp. Clin. Cancer Res.* **2019**, *38*, 58.
2. Cirillo, F.; Pellegrino, M.; Malivindi, R.; Rago, V.; Avino, S.; Muto, L.; Dolce, V.; Vivacqua, A.; Rigiracciolo, D.C.; De Marco, P.; et al. GPER Is Involved in the Regulation of the Estrogen-Metabolizing CYP1B1 Enzyme in Breast Cancer. *Oncotarget* **2017**, *8*, 106608–106624.
3. Schuermann, A.; Helker, C.S.M.; Herzog, W. Angiogenesis in Zebrafish. *Semin. Cell Dev. Biol.* **2014**, *31*, 106–114.
4. Cai, W.; Sakaguchi, M.; Kleinridders, A.; Gonzalez-Del Pino, G.; Dreyfuss, J.M.; O'Neill, B.T.; Ramirez, A.K.; Pan, H.; Winnay, J.N.; Boucher, J.; et al. Domain-Dependent Effects of Insulin and IGF-1 Receptors on Signalling and Gene Expression. *Nat. Commun.* **2017**, *8*, 14892.
5. Pandini, G.; Medico, E.; Conte, E.; Sciacca, L.; Vigneri, R.; Belfiore, A. Differential Gene Expression Induced by Insulin and Insulin-like Growth Factor-II through the Insulin Receptor Isoform A. *J. Biol. Chem.* **2003**, *278*, 42178–42189.
6. Pearce, D.A.; Nirmal, A.J.; Freeman, T.C.; Sims, A.H. Continuous Biomarker Assessment by Exhaustive Survival Analysis. *bioRxiv* 2018, 208660.

Supplementary data

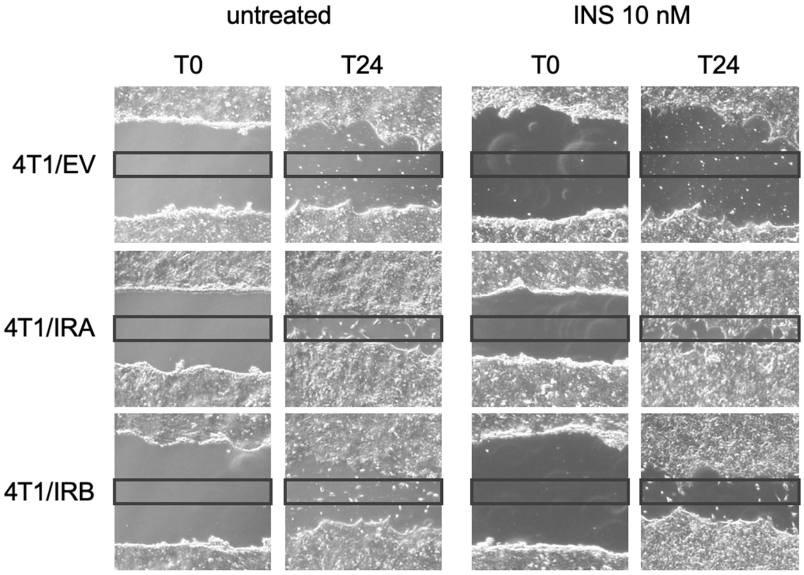

Figure S1. Cell migration evaluated by wound-healing assay. 4T1/EV, 4T1/IR-A and 4T1/IR-B were treated with or without 10 nM of insulin. Black lines indicate the wound borders at the beginning of the assay and recorded 24 h post-scratching.

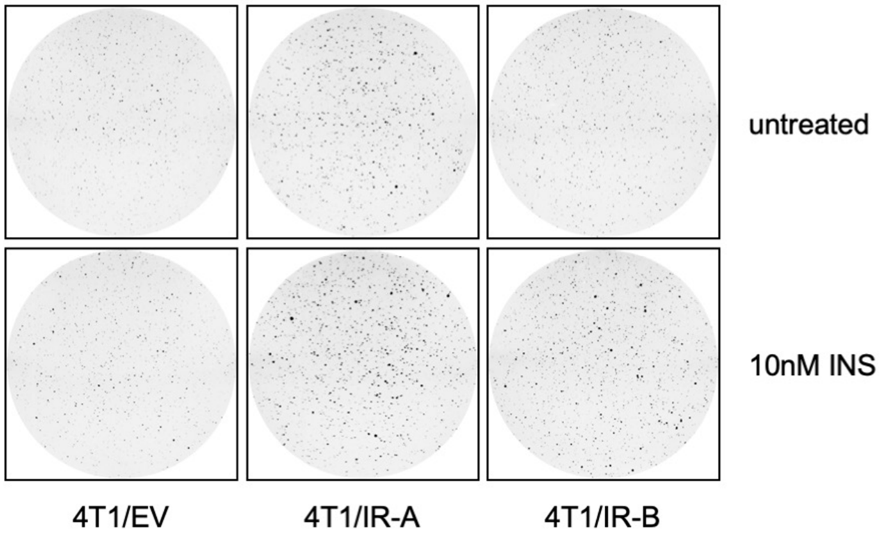

Figure S2. Colony formation. Cells were seeded in soft agar and grown in 5% charcoal-stripped serum for 3 weeks and then treated or not with 10 nM of insulin. Colonies were stained with MTT and then photographed.

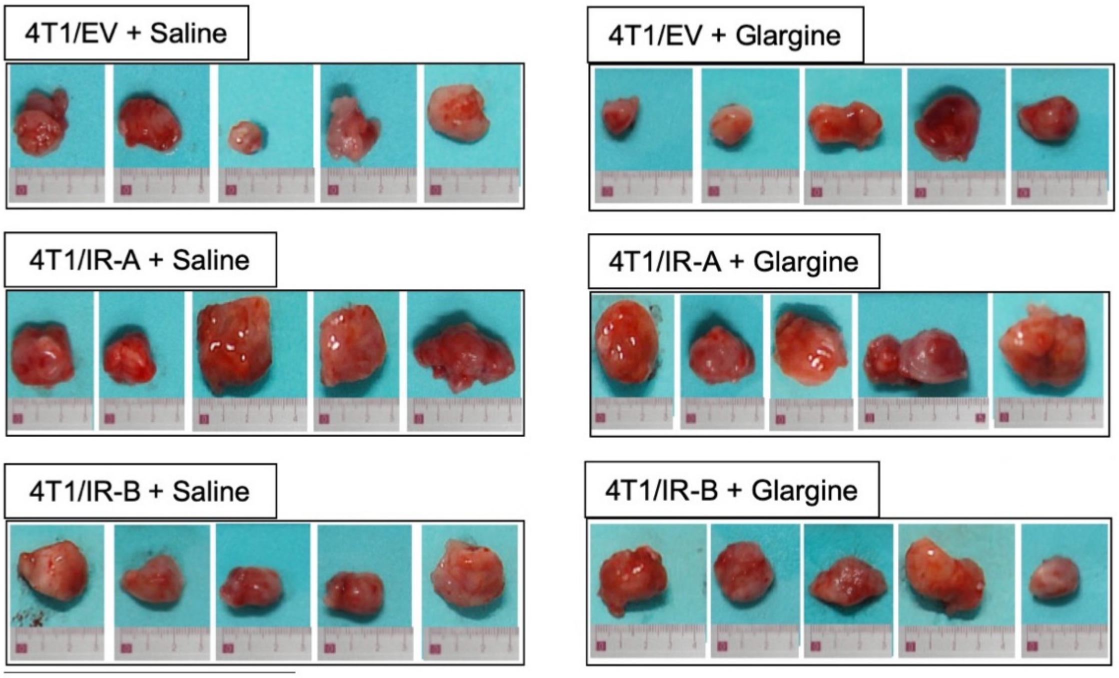

Figure S3. Tumor growth in nude mice. Gross appearance of tumors obtained from 4T1/IR-A, 4T1/IR-B cells, compared to 4T1/EV inoculated mice, treated or not with insulin glargine.

Additional files

Additional file 1 (.xlsx). ***Dysregulated transcripts***. Excel file containing the list of dysregulated transcripts for each comparison and their statistics. Each comparison is reported in a separate sheet of the Excel file.

Additional file 2 (.xlsx). ***Dysregulated pathways***. Excel file containing the dysregulated pathways for each comparison. The values that are shown for each pathway are the corrected accumulators calculated by the MITHrIL algorithm. The corrected accumulators of pathways that were not found statistically dysregulated were set as 0 (no deregulation).

Additional file 3 (.xlsx). **MITHrIL results**. Excel file containing the unprocessed MITHrIL results with both statistically significant dysregulated pathways and not.

Mendeley data access for:
additional file 1
additional file 2
additional file 3

https://data.mendeley.com/datasets/hg3nvm2ktj/draft?a=3644d5cb-dea9-42b7-b3d1-8ed5ffa44b46
